# Role of Serum Interleukin-6, Interleukin-1β and Interleukin-10 in Assessment of Disease Activity and Nutritional Status in Patients with Inflammatory Bowel Disease

**DOI:** 10.3390/jcm12185956

**Published:** 2023-09-13

**Authors:** Małgorzata Godala, Ewelina Gaszyńska, Konrad Walczak, Ewa Małecka-Wojciesko

**Affiliations:** 1 Department of Nutrition and Epidemiology, Medical University of Lodz, 90-752 Lodz, Poland; ewelina.gaszynska@umed.lodz.pl; 2 Department of Internal Medicine and Nephrodiabetology, Medical University of Lodz, 90-549 Lodz, Poland; konrad.walczak@umed.lodz.pl; 3 Department of Digestive Tract Diseases, Medical University of Lodz, 90-419 Lodz, Poland; ewa.malecka-panas@umed.lodz.pl

**Keywords:** inflammatory bowel disease, Crohn’s disease, ulcerative colitis, Il-10, Il-6, IL-1β

## Abstract

Inflammatory bowel diseases (IBD) are characterised by multifactorial and chronic inflammation. Much attention has been paid to immune dysfunction in inflammatory bowel diseases. The aim of this study was to assess the usefulness of serum IL-6, IL-1β and IL-10 in determining the activity and nutritional status in IBD patients. The case–control study was carried out on 82 patients with IBD; the control group consisted of 25 clinically healthy subjects. The serum concentrations of IL-6, IL-1 β and IL-10 were determined by the quantitative sandwich enzyme-linked immunosorbent assay. There were no significant differences in IL-6 and IL-1β levels in UC and CD patients according to disease activity as assessed by the Montreal classification, Partial Mayo Score and CDAI. Significantly higher IL-6 levels were found in patients with low body fat in comparison to patients with normal body fat. Furthermore, significantly higher mean IL-6 levels were observed in patients with excess body fat in comparison to patients with normal body fat, and also in comparison to patients with deficient body fat. IL-6 and IL-1β may provide extra information regarding the nutritional status of IBD patients. IL-10 can be considered a non-invasive biomarker of IBD activity.

## 1. Introduction

Inflammatory bowel diseases (IBDs) are characterised by a multifactorial, as yet not fully understood aetiology and a chronic course with periods of remission and exacerbation. Data gathered over the last few decades reveal that prevalence of inflammatory bowel disease is increasing [1,2,3]. Recent data have shown that IBD affects nearly 100,000 people in Poland, with 23,574 patients with Crohn’s disease (CD) and 73,235 with ulcerative colitis (UC), across all age groups, but the diagnosis is most often made in the third decade of life [1]. The chronic nature of IBD, which is associated with long-term pharmacotherapy, multiple hospitalisations as well as surgical treatment, is a cause of work incapacity and disability [1,2,3,4,5,6,7].

The aetiopathogenesis of IBD is multifactorial. The development of IBD is thought to be influenced by complex interactions between environmental and bacterial factors, genetic predisposition and the disruption of intestinal immune mechanisms [7,8,9,10,11,12,13,14,15,16,17]. Much attention has been paid to immune dysfunction in IBD [3,11]. It has been observed that CD is dominated by the activation of Th1 cell subpopulations [15], which release large amounts of TNF, IFN, IL-2, -6 and -8. In contrast, UC has been shown to activate both Th1 and Th2 cells [17], which is associated with the increased production of immunomodulatory cytokines IL-4, -5, -10 and TNF-α, which support the humoral response by inducing immunoglobulin synthesis. Importantly, there is a group of cytokines, such as IL-1β and IL-6, that play a role in the pathogenesis of both forms of IBD. These substances activate the secondary response of Th1 and Th2 lymphocytes, as a result of the stimulation of immune cells such as macrophages [18].

Interleukin 1 (IL-1) comprises a family of cytokines that includes more than 10 molecules, e.g., IL-1β. IL-1 is one of the main regulators of the inflammatory response, secreted mainly by monocytes and macrophages of various tissues in response to bacterial wall lipopolysaccharide [19,20]. IL-1β is mainly present in its secretory form, almost always released by peripheral blood monocytes and cells isolated from the inflamed gastrointestinal mucosa of IBD patients. IL-1β initiates and exacerbates the inflammatory process in the colon [17,21].

IL-6 is a pro-inflammatory cytokine with pleiotropic effects [22,23]. IL-6 is one of the main factors regulating the body’s defence mechanisms. Together with IL-1β, it initiates and intensifies the inflammatory process in the colon due to its properties stimulating the secretion of other inflammatory mediators (e.g., IL-8 and eicosanoids) [23,24]. The continued stimulation and maintenance of this process damages tissues through the action of released proteolytic enzymes and oxygen free radicals [24,25].

IL-10 is an anti-inflammatory cytokine secreted by Th2 lymphocytes [26,27,28,29]. Studies have shown that mice deficient in IL-10 secretion developed chronic colitis and mucosal proliferation [29]. It was further observed that IL-10 can reduce inflammation in animals and in vitro models, suggesting that it plays a role in reducing Th1-mediated mucosal inflammation [26,29]. Impaired IL-10 production was also noted in both UC and CD [27,28,30].

Generalised inflammation in IBD patients, being a result of excessive cytokine production, leads to a series of metabolic reactions that result in, among others, loss of appetite, increased energy consumption, as well as protein and fat breakdown. This results in the loss of muscle and fat mass, which causes malnutrition in patients with IBD [22,25,31]. Malnutrition is a common problem in IBD patients. It makes therapy less successful and worsens prognosis [1,2,32].

Endoscopy combined with biopsy is the most effective way to diagnose IBD. This method is effective but also invasive and causes discomfort to patients. Therefore, alternative non-invasive biomarkers to assess disease activity are being sought. The aim of this study was to assess the usefulness of serum IL-6, IL-1β and IL-10 in determining the activity and nutritional status in IBD patients.

## 2. Material and Methods

### 2.1. Study and Control Groups

In total, 82 patients with IBD (48 patients with CD and 34 patients with UC), recruited from the Medical University of Lodz, participated in the study. The diagnosis of IBD was made on the basis of clinical image, endoscopic, histopathological and imaging studies. Obese patients do not have comorbidities such as diabetes or metabolic syndrome. The control group consisted of 25 healthy volunteers. All studied participants were asked about smoking habit and family history of IBD.

The study was conducted in accordance with the Declaration of Helsinki, and approved by the Bioethics Committee of the Medical University of Lodz (No. RNN/70/22/KE, 14 June 2022). All the subjects gave their written consent to participate in the study.

### 2.2. Disease Activity

IBD activity was assessed using validated scales. For patients with CD, the Crohn’s Disease Activity Index (CDAI) and the Montreal classification were used [33]. For patients with UC, the Partial Mayo Score and the Montreal classification were used [34].

### 2.3. Nutritional Status

All subjects had their waist circumference measured and BMI determined. The body composition of the subjects was assessed by Bioelectrical Impedance Analysis (BIA).

### 2.4. Serum Markers of the Nutritional Status

The serum concentrations of IL-6, IL-1 β and IL-10 were determined by the quantitative sandwich enzyme-linked immunosorbent assay.

### 2.5. Statistical Analysis

Statistical analysis was performed with the use of Statistica™ 14 software (TIBCO Software Inc., Palo Alto, CA, USA). The normality of distribution was checked using the Shapiro–Wilk W test. In univariate analyses, the Mann–Whitney U test was used for the dichotomous grouping of variables, or the Kruskal–Wallis H test when a grouping variable had more than two categories. Correlations were assessed with the Spearman’s coefficient (r). A level of *p* < 0.05 was considered statistically significant.

## 3. Results

### 3.1. Study Characteristics

The characteristics of the study group are shown in Table 1. In total, 42 women (51.2%) and 40 men (48.8%) with IBD participated in the study. Among the patients, 40 subjects (48.8%) had tertiary education and 14 patients (17.1%) smoked cigarettes. Of all patients, 66 subjects (80.5%) received biological treatment (infliximab, vedolizumab), 64 (78.0%) received 5-aminosalicylic acid preparations, 33 patients (40.2%) received immunosuppressants (azathioprine) and 25 subjects (30.5%) were administered corticosteroids (prednisone). Nearly half of the patients (48.8%) were in the period of clinical remission and did not demonstrate disease symptoms.

No differences were found between patients with IBD and the control group in terms of age, gender and smoking. Furthermore, BMI or waist circumference values were not different in the above groups. However, significant differences were found in the body compositions of the subjects. Patients with IBD demonstrated significantly lower mean contents of body fat.

### 3.2. Inflammatory Markers in CD and UC

The authors did not find any significant differences in mean Il-6 or IL-1β levels between IBD patients and controls. Significant differences were noted for Il-10, the concentration of which was significantly higher in the UC group than in the control group (19.83 pg/mL vs. 5.30 pg/mL, *p* = 0.0096), but did not differ significantly between patients with UC and CD or between patients with CD and the control group (Table 2).

There were no differences in the median levels of the cytokines studied by age of the subjects, place of residence, and smoking. In addition, the median IL-1β concentration was significantly higher in the group of men with IBD than in the group of women with IBD (3.0 pg/mL vs. 2.8 pg/mL, *p* = 0.0422) and in the group of patients with a positive family history (3.1 pg/mL vs. 2.9 pg/mL, *p* = 0.0437). IL-6 levels were significantly higher in patients with IBD with a positive family history in comparison to those without a family history of inflammatory bowel disease (6.3 pg/mL vs. 5.2 pg/mL, *p* = 0.0197).

### 3.3. Inflammatory Markers and Disease Activity

There were no significant differences in IL-1 β and IL-10 levels in IBD patients according to the administration of biological treatment and past intestinal resections. However, patients suffering with the disease for more than 10 years demonstrated significantly higher levels of IL-1β and IL-10 in comparison to other patients (9.6 pg/mL vs. 2.9 pg/mL, *p* = 0.030 and 14.7 pg/mL vs. 2.5 pg/mL, *p* = 0.031, respectively). The mean IL-6 levels were found to be significantly lower in patients undergoing biological treatment in comparison to other patients (5.1 pg/mL vs. 6.6 pg/mL, *p* = 0.0067, respectively) (Table 3).

There were no significant differences in IL-6 and IL-1β levels in UC patients according to disease activity as assessed by the Montreal classification and Partial Mayo Score. Significantly higher IL-10 levels were found in patients in remission in comparison to patients with moderate and severe exacerbations of symptoms (the Partial Mayo Score 0 vs. 2 and 3; 23.6 pg/mL vs. 3.4 pg/mL vs. 2.2 pg/mL, respectively, *p* = 0.0059). Besides this, higher IL-10 levels were noted in patients with asymptomatic and mild disease flares in comparison to patients with moderate and severe disease flares according to the Montreal classification (24.0 pg/mL vs. 14.7 pg/mL vs. 2.4 pg/mL vs. 2.0 pg/mL, respectively, *p* = 0.0022) (Table 4). There were no significant differences in IL-6 and IL-1β levels in patients with CD according to disease activity, assessed with the Montreal classification and CDAI. Higher IL-10 levels were found in patients in remission (CDAI < 150) in comparison to patients with mild, moderate and severe symptoms (8.5 pg/mL vs. 1.8 pg/mL vs. 2.3 pg/mL vs. 2.3 pg/mL, respectively, *p* = 0.0462). No differences were observed in the mean Il-10 levels according to the degree of extension of Crohn’s disease lesions (Table 5).

### 3.4. Inflammatory Markers and Nutritional Status of Patients

There were no differences in mean serum levels of IL-6, IL-1β and IL-10 according to BMI and waist circumference.

Significantly higher levels of IL-6 and IL-1β were observed in patients who admitted a significant weight loss in the last six months in comparison to patients with a weight loss of less than 10% of the baseline body weight (5.9 pg/mL vs. 4.9 pg/mL, *p* = 0.048 and 3.0 pg/mL vs. 2.9 pg/mL, *p* = 0.049, respectively). Furthermore, IL-1β levels positively correlated with muscle mass (rho = 0.2427, *p* = 0.0280) and lean body mass (rho = 0.2668, *p* = 0.0154) in patients with IBD.

Significantly higher IL-6 levels were found in patients with low body fat in comparison to patients with normal body fat (5.7 pg/mL vs. 4.6 pg/mL, *p* = 0.0102). Furthermore, significantly higher mean IL-6 levels were observed in patients with excess body fat in comparison to patients with normal body fat (6.2 pg/mL vs. 4.6 pg/mL, *p* = 0.0415), and also in comparison to patients with deficient body fat (6.2 vs. 5.7, respectively, *p* = 0.0078) (Table 6).

### 3.5. Correlations between Inflammatory Markers

In all participants, IL-10 levels positively correlated with IL-6 (r = 0.2616, *p* < 0.001) and IL-1β (r = 0.3308, *p* < 0.0001). Additionally, in all participants, IL-6 levels positively correlated with IL-1β levels (r = 0.3238, *p* < 0.0001). In CD patients, IL-10 levels positively correlated with IL-6 (r = 0.4129, *p* < 0.0001) and IL-1β (r = 0.3914, *p* < 0.001) levels. Furthermore, in the CD group, IL-6 levels positively correlated with IL-1β levels (r = 0.3155, *p* < 0.001). In the UC group, no significant correlations were found between the studied cytokines (Table 7).

## 4. Discussion

In our study, we are seeking easily determined biomarkers that may be helpful in assessing nutritional status and IBD activity. Due to the chronic inflammation that occurs in IBD patients, we assessed the usefulness of determining IL-6, IL-1 β and IL-10 levels.

In our study, IL-6 levels did not differ significantly between patients with CD, UC and controls. There was also no correlation between IL-6 levels and disease activity in either the CD or UC groups. The results of other available studies are inconclusive.

A study by Polinska et al. found that the overexpression of IL-6 was noted only in the inflamed mucosa and correlated with disease activity in patients with UC. The authors confirmed its significantly lower levels in UC patients in remission in comparison to patients with active IBD [35].

In a study by Sobolewska et al., IL-6 levels did not differ significantly between patients with CD and UC, and this observation was similar to that in our study. In contrast, they found higher IL-6 levels during clinical exacerbations [36].

Similar data were obtained in a study conducted by Wędrychowicz, in which IL-6 levels increased in the serum of patients with active UC and decreased during remission. Furthermore, IL-6 levels correlated with the severity and extension of inflammatory bowel lesions. IL-6 was also shown to be present in the stool in both the active and inactive phases of UC [37].

In contrast, in a study by Matusiewicz et al., IL-6 levels positively correlated with disease activity in both UC and CD patients [38].

IL-6 strongly stimulates inflammatory processes in a direct manner. In contrast, other pro-inflammatory cytokines, particularly interleukin 1, are factors that stimulate IL-6 production [39]. These data are confirmed by results of our study, in which IL-6 levels positively correlated with IL-1β levels.

On the other hand, we did not confirm significant differences in IL-6 levels between IBD patients and healthy subjects. This was probably affected by the use of biological therapy, which the majority of IBD patients received [39,40]. The administration of biological drugs, especially infliximab, affects the levels inflammatory parameters, including the concentration of pro-inflammatory cytokines [18,41,42,43,44]. Infliximab inhibits TNF-α activity in in vitro bioassays (44lubecka-macura). In vivo, it rapidly forms stable complexes with human TNF-α, which results in a loss of biological activity caused by TNF-α [39,40]. Thus, this treatment reduces the influx of inflammatory cells into the affected areas of the gut and reduces the amount of inflammatory markers. Not only does TNF-α directly affect immune cell function but it also increases the synthesis of cytokines, including IL-6. As a result, IL-6 levels get reduced [39,41]. The effect of diminishing IL-6 levels after biological treatment was confirmed in our study. In fact, we demonstrated significantly lower IL-6 levels in patients receiving biological therapy in comparison to other patients. An implementation of biological treatment and, as a consequence, a decrease in IL-6 levels in these patients possibly explains why there are no differences in mean IL-6 levels between patients and healthy controls.

The present study also assessed IL-1β levels in patients with IBD. Similarly to IL-6, IL-1β levels did not appear to be associated with disease activity in patients with UC and CD, and did not differ significantly from those observed in healthy subjects.

Different data were obtained in a study by Wędrychowicz et al., in which elevated IL-1β levels were found in the stool and serum of patients with UC, especially in the active phase of the disease [37,45]. However, in this study it was noted that the levels of IL-1β secreted into the intestinal lumen did not correlate with systemic inflammatory markers (leukocytosis and increased platelet count) due to its short half-life and accelerated degradation by proteolytic enzymes [45]. This may explain the lack of change in IL-1β levels between healthy subjects and sick patients in the period of symptom exacerbation and remission in our study.

In our study, in addition to pro-inflammatory cytokines, IL-10 levels were also assessed in patients with IBD. IL-10 appeared to have an inhibitory effect on the synthesis of pro-inflammatory cytokines released from T lymphocytes and macrophages in active inflammatory bowel disease, and the administration of IL-10 to patients with UC reduces their complaints [46,47,48,49]. In our study, we found significantly higher IL-10 levels in patients with UC in comparison to healthy controls. However, we did not observe significant differences between patients with UC and CD, or between patients with CD and controls. Importantly, IL-10 levels were associated with disease activity only in UC. Higher levels of this cytokine were noted in patients in remission and with mild flares in comparison to those with moderate and severe disease. Similar data were obtained by other authors.

A study by Wozniak-Stolarska showed elevated serum IL-10 levels in patients with IBD in comparison to healthy subjects [50], and Melgar et al. observed increased IL-10 mRNA levels in the intestinal mucosa of patients with UC in comparison to healthy subjects. This study showed that IL-10 levels correlated with disease activity, and higher levels were observed in patients in remission [51].

The results of a meta-analysis of eight studies evaluating IL-10 levels in patients with IBD, conducted in 2019, appear to be consistent with the results obtained in our study. The meta-analysis showed higher IL-10 levels in patients with UC in comparison to healthy subjects, while these levels did not differ between patients with UC and CD. Furthermore, it was confirmed that increased IL-10 levels contribute to the pathogenesis and progression of UC [52].

The increase in IL-10 levels in IBD patients, especially during remission, is probably due to the body’s response to the increased inflammation that occurs in CD and UC, and an attempt to reduce it. It seems that the inclusion of new, more sensitive markers in the diagnosis and follow-up of patients, in addition to non-specific, standard markers of inflammatory process, would enable one to better assess the patient’s condition and prognosis, and help in choosing optimal therapy. IL-10 deserves special attention in this group.

We further investigated the relationship between the levels of the studied cytokines and the nutritional status of patients with IBD. We found significantly higher mean IL-6 levels among IBD patients with excess body fat. However, we did not observe significant differences in IL-6 levels according to BMI values.

No papers were found that assessed the relationship between IL-6 and the nutritional status of IBD patients. In contrast, the relationship of increased inflammatory processes with obesity has long been discussed in the literature [53,54,55]. A lot of studies have shown that obese individuals demonstrate an increased production of adipokines that promote the development of metabolic diseases related to obesity. These include IL-6 [53,55,56]. The secretory activity of adipose tissue changes and depends on its cellular composition. In addition to adipocytes, adipose tissue contains preadipocytes, lymphocytes, macrophages, mast cells, eosinophils and fibroblasts [55,56]. In obesity, there is a significant influx of macrophages into adipose tissue, which is associated with the development of inflammation. Macrophages in adipose tissue are characterised by a different phenotype and different actions. In the adipose tissue of slim individuals, alternatively activated type II macrophages (macrophages type II, M2) are found, whereas in obese individuals, classically activated type I macrophages (macrophages type I, M1) predominate [54]. Type M1 macrophages are responsible for the development of inflammation. They are activated by cytokines produced by Th1 lymphocytes, which release interferon gamma (IFN-γ). Stimulated M1 macrophages produce pro-inflammatory cytokines, including IL-6 [56]. The results of our study support these observations.

In contrast, in our study, we showed increased IL-6 levels also in subjects with body fat deficiency and reporting a significant weight loss in the last six months. Subjects with body fat deficiency had higher mean IL-6 levels in comparison to patients with normal body fat, but lower levels than patients with excess body fat.

Studies have shown a relationship between IL-6 and eating disorders manifesting with emaciation and body composition abnormalities, including extremely low levels of body fat. In a study conducted by Ziora et al., IL-6 levels were assessed in a group of girls with anorexia nervosa. Mean serum IL-6 levels in patients did not differ significantly from mean levels of this cytokine in a group of healthy girls. In contrast, IL-6 levels were significantly lower in patients with fat deficiency than in the group of obese girls [57]. This relationship was not confirmed by our own study, in which IL-6 levels were significantly higher in the body fat-deficient group than in subjects with normal levels of body fat. It is possible that in underweight patients, high IL-6 levels were related to a great loss of body weight and changes in the body composition. Inflammatory parameters that are observed in IBD are altered in the event of significant reductions in body weight, resulting in reductions in body fat levels [58,59]. 

In our study, we showed that IL-1β concentration positively correlates with muscle mass content in patients with IBD. However, we did not demonstrate a correlation between IL-1β concentration and the body fat content or BMI of the subjects. The results of studies carried out by other authors on IL-1β values in relation to muscle mass content are inconclusive.

In a study conducted by Ziora et al., patients with mental anorexia and low muscle mass exhibited higher levels of IL-1β than subjects with normal nutritional status [57].

Different data were obtained in a study by Allende et al., in which no significant differences in IL- 1β levels were found in subjects with mental anorexia and in healthy subjects with normal body composition, including normal muscle mass [60].

Our results suggest an association between IL-1β levels and the nutritional status of IBD patients in relation to muscle mass. Changes in body composition, i.e., a decrease in skeletal muscle and adipose tissue mass, resulting in progressive weight loss, are frequently observed in patients with IBD [61,62,63,64,65]. IL-1β is implicated as a cytokine in the development of anorexia associated with chronic disease (most commonly cancer), probably mediated through elevated levels of corticotropin-releasing hormone, which inhibits food intake [66,67]. Furthermore, IL-1β can directly stimulate lipolysis, contributing thereby to weight loss and changes in body composition [68].

Our study has some limitations. It was conducted in a small group of patients. In addition, the majority of participants were directed towards biological treatment, which impacts inflammatory parameters. Therefore, serum cytokine measurements in these patients should be interpreted with caution.

In light of our study, the assessed biomarkers may be useful in controlling the activity of IBD and the nutritional status of patients, which is crucial for the success of therapy. Our results suggest a relationship between elevated IL-6 levels and the nutritional status of IBD patients, especially those with abnormal body fat. We did not show a correlation between IL-6 levels and BMI, which indicates a need to assess body composition in these patients. BMI does not verify body fat content, which is crucial in assessing the nutritional status and relevant to ongoing inflammatory processes. Furthermore, we showed an association between IL-6 and IL-1β levels and non-intentional weight reduction in patients. Thus, the determination of IL-6 and IL-1β levels in patients with IBD may provide extra information regarding the anthropometric assessment of their nutritional status.

Furthermore, we found a relationship between IL-10 levels and disease activity in IBD patients. Higher mean levels were observed in patients in remission and with a mild course. In contrast, we did not confirm a relationship between IL-10 levels and nutritional status in IBD patients, regardless of the applied method. From our observations, we can claim that inflammation reduces IL-10 levels.

## 5. Conclusions

IL-6 and IL-1β levels appeared to be predictors of changes in body composition, and IL-6 alone is also a predictor of body fat content in IBD patients, regardless of the commonly used markers of nutritional status, such as BMI or waist circumference. They may provide extra information regarding the nutritional status of these patients. In light of our study, IL-10 can be considered a non-invasive biomarker of IBD activity.

## Figures and Tables

**Table 1:** General characteristics of study participants.

	IBD N(%)/Mean ± SD	Controls N(%)/Mean ± SD
CD	48 (58.5)	-
UC	34 (41.5)	-
Age (years)	38.1 ± 11.6	33.6 ± 9.1
Female	42 (51.2)	15 (60)
Level of education		
Secondary	42 (51.2)	4 (16) *
High	40 (48.8)	21 (84) *
Current smoking	14 (17.1)	3 (12)
Disease duration	8.4 ± 5.7	-
Past intestinal resection	22 (26.8)	-
Weight lost during last 6 months (%)	50 (60.9)/16.5 ± 8.2	-
Anthropometry		
BMI (kg/m^2^)	24.23 ± 4.76	24.64 ± 3.97
<18.5	10 (12.2)	3 (12)
18.5–24.9	38 (46.3)	11 (44)
25.0–29.9	26 (31.7)	9 (36)
>30.0	8 (9.8)	2 (8)
Waist circumference (cm)	88.9 ± 14.5	85.3 ± 9.1
Normal	57 (69.5)	18 (72)
High	25 (30.5)	7 (28)
Fatty tissue (%)	27.1 ± 9.6	30.1 ± 8.6 *
Low	14 (17.1)	2 (8) *
Normal	59 (71.9)	15 (60) *
High	9 (11.0)	8 (32) *
Free fat mass (%)	72.1 ± 11.2	69.9 ± 8.6 *
Water (%)	55.1 ± 6.5	53.8 ± 4.1
Low	26 (31.7)	10 (40) *
Normal	56 (68.3)	15 (60) *
Muscle mass (kg)	43.4 ± 13.7	43.6 ± 9.6
Medications		
Biological therapy	66 (80.5)	-
Immunosuppression	33 (40.2)	-
Steroids	25 (30.5)	-
5-ASA	64 (78.0)	-
Disease activity		
Self-reported clinical stage of disease		-
Remission	40 (48.8)	-
Moderate	27 (32.9)	-
Active	15 (18.3)	-
UC		
Partial Mayo Score (0/1/2/3)	17 (50.0)/0 (0)/11 (32.4)/6 (17.6)	-
Montreal classification		
E1/E2/E3	4 (11.8)/16 (47.0)/14 (41.2)	-
S0/S1/S2/S3		-
	12 (35.3)/10 (29.4)/11 (32.4)/3 (8.8)	
CD		
CDAI (0/1/2/3)	17 (35.4)/10 (20.9)/17 (35.4)/4 (8.3)	-
Montreal classification		-
A1/A2/A3	8 (16.7)/36 (75)/4 (8.3)	
L1/L2/L3	17 (35.4)/7 (14.6)/24 (50)/0 (0)	-
B1/B2/B3	21 (43.8)/18 (37.5)/15 (3.2)	-

* *p* < 0.05.

**Table 2:** Concentrations of IL-6, IL-1 β and IL-10 in the study participants.

Analyzed Marker	Study Group	Statistical Parameter					*p*-Value *
		Mean	SD	Median	Q_1_–Q_3_	Min.–Max.	
Il-6 (pg/mL)	CD	13.46	21.16	5.90	3.85–7.20	0.40–96.90	0.7484
	UC	19.20	49.34	5.65	4.40–7.40	0.60–272.40	
	Control	15.68	14.16	8.80	4.70–27.60	4.10–42.70	
Il-1 beta (pg/mL)	CD	11.61	18.23	2.92	2.77–9.91	1.65–82.29	0.9038
	UC	12.51	16.98	2.94	2.26–14.92	0.89–57.89	
	Control	10.16	11.19	3.17	2.77–21.98	1.91–30.40	
Il-10 (pg/mL)	CD	13.89	19.62	2.31	1.90–20.09	0.73–68.95	0.0063 **
	UC	19.83	24.14	8.05	2.25–24.05	1.47–90.10	
	Control	5.30	6.60	3.10	1.80–5.10	0.72–25.20	

* Statistical significance for the model used. All between-group comparisons were controlled for the patients’ age and gender. ** Post-hoc multiple comparisons. Il-10, CD vs. UC *p* = 0.4132; UC vs. controls *p* = 0.0096; CD vs. controls *p* = 0.1270.

**Table 3:** Concentrations of IL-6, IL-1 β and IL-10 according to basic clinical characteristics of patients with IBD.

	IL-6 (pg/mL) Me [Q1–Q3]	IL-1β (pg/mL) Me [Q1–Q3]	IL-10 (pg/mL) Me [Q1–Q3]
Disease duration			
< 5	5.9 [3.9–7.4]	2.9 [2.7–3.0]	2.1 [1.9–11.6]
5–10	5.2 [4.6–7.4]	2.9 [2.2–3.0]	2.5 [2.1–23.9]
>10	5.9 [3.9–7.0]	9.6 [2.8–39.4]	14.7 [2.2–48.0]
*p*-value	0.9533	0.0300 * 0.0011 **	0.0314 *
Biology therapy			
YES	5.1 [4.4–6.9]	2.9 [2.6–12.2]	2.5 [1.9–23.9]
NO	6.6 [4.0–15.0]	2.9 [2.8–18.6]	2.5 [2.1–11.9]
*p*-value	0.0067 *	0.6314	0.8699
Surgery			
YES	5.7 [3.9–6.3]	2.8 [2.4–3.1]	2.3 [2.0–14.7]
NO	5.9 [4.4–7.5]	2.9 [2.7–12.7]	2.7 [2.0–23.7]
*p*-value	0.7894	0.3708	0.3002

* Univariate analyses. Post-hoc multiple comparisons. IL-1β, <5 vs. 5–10 NS; <5 vs. >10 *p* = 0.2185; 5–10 vs. >10 *p* = 0.0291. IL-10, <5 vs. 5–10 *p* = 0.3316; <5 vs. >10 *p* = 0.0280; 5–10 vs. >10 NS. ** A multivariate analysis, involving all the investigated factors, controlled for age and gender.

**Table 4:** Concentrations of IL-6, IL-1 β and IL-10 according to UC activity.

	IL-6 (pg/mL) Me [Q1–Q3]	IL-1β (pg/mL) Me [Q1–Q3]	IL-10 (pg/mL) Me [Q1–Q3]
Partial Mayo Score			
0	5.3 [4.4–7.4]	2.9 [2.6–37.4]	23.6 [2.3–48.6]
2	6.0 [5.4–6.9]	3.0 [2.1–26.2]	3.4 [2.3–14.7]
3	4.4 [3.7–7.7]	2.8 [2.3–3.0]	2.2 [1.9–2.6]
*p*-value	0.2860	0.370	0.0059 *
Montreal Classification			
E1	5.6 [5.1–59.9]	7.9 [2.3–27.4]	35.0 [19.4–56.8]
E2	5.9 [4.6–6.9]	2.9 [2.1–12.2]	2.6 [2.2–23.6]
E3	5.9 [3.7–7.7]	3.0 [2.8–26.2]	2.6 [2.1–17.4]
*p*-value	0.8337	0.8563	0.1118
			
S0	6.0 [4.6–53.9]	2.9 [2.3–41.7]	24.0 [2.6–46.4]
S1	4.8 [4.4–5.3]	3.0 [2.8–12.2]	14.7 [2.1–19.8]
S2	5.9 [4.5–6.9]	3.0 [2.2–26.2]	2.4 [2.2–11.6]
S3	7.7 [7.7–7.7]	2.6 [2.3–2.9]	2.0 [1.5–2.6]
*p*-value	0.1130	0.8013	0.0022 *

* Univariate analyses. Post-hoc multiple comparisons. Il-10, Mayo Score 0 vs. 2 *p* = 0.0373; 0 vs. 3 *p* = 0.0099322233243; 2 vs. 3 *p* =0.6219. Il-10, S0 vs. S1 *p* = 0.1769; S0 vs. S2 *p* = 0.0083; S0 vs. S3 *p* = 0.0675; S1 vs. S2 *p* = 0.0090; S1 vs. S3 *p* = 0.0292; S2 vs. S3 *p* = 0.7779.

**Table 5:** Concentrations of IL-6, IL-1 β and IL-10 according to Montreal classification of CD activity.

	IL-6 (pg/mL) Me [Q1–Q3]	IL-1β (pg/mL) Me [Q1–Q3]	IL-10 (pg/mL) Me [Q1–Q3]
Age at onset (years)			
A1 < 16	6.1 [4.9–6.6]	3.0 [2.8–3.5]	17.1 [2.2–34.1]
A2 17–40	5.9 [3.8–7.4]	2.9 [2.7–3.1]	2.2 [1.8–13.6]
A3 > 40	4.0 [3.0–50.8]	2.9 [2.8–25.5]	2.1 [1.8–33.2]
*p*-value	0.6631	0.5373	0.3924
Localization			
L1 Ileum	5.0 [3.8–6.5]	2.9 [2.9–9.6]	2.2 [1.9–13.6]
L2 Colon	6.6 [4.9–7.6]	2.4 [2.0–2.9]	11.4 [1.8–37.0]
L3 Ileum + colon	5.9 [3.8–6.6]	2.9 [2.8–10.9]	2.3 [1.8–19.5]
*p*-value	0.5291	0.2207	0.7955
Course of the disease			
B1 No stenoses or fistulas	5.9 [3.9–7.4]	2.9 [2.7–10.2]	8.6 [1.9–37.0]
B2 Stenoses	5.7 [4.4–6.3]	2.9 [2.8–3.0]	2.2 [1.8–2.3]
B3 Fistulas	6.1 [5.5–7.6]	2.9 [2.7–3.0]	2.2 [1.8–8.0]
Perianal lesions	6.1 [2.6–9.0]	2.7 [2.4–14.1]	11.2 [2.1–28.5]
*p*-value	0.6029	0.9337	0.1095
CDAI			
<150	5.9 [3.9–18.8]	3.0 [2.9–19.1]	8.5 [1.9–32.0]
150–220	6.3 [4.7–7.4]	2.9 [2.3–3.0]	1.8 [1.7–2.9]
221–450	4.9 [3.3–6.1]	2.9 [2.7–2.9]	2.3 [2.0–19.5]
>450	5.9 [5.9–35.2]	2.8 [2.6–2.9]	2.3 [1.9–2.6]
*p*-value	0.3000	0.2810	0.0462 *

* A univariate analysis. Post-hoc multiple comparisons. Il-10, CDAI < 150 vs. CDAI 150–220, *p* = 0.0373; CDAI < 150 vs. CDAI 221–450, *p* = 0.0129; CDAI < 150 vs. CDAI > 450, *p* = 0.0034; CDAI 150–220 vs. CDAI 221–450, *p* = 0.9732; CDAI 150–220 vs. CDAI > 450, *p* = 0.2332; CDAI 221–450 vs. CDAI > 450, *p* =0.5239.

**Table 6:** Concentrations of IL-6, IL-1 β and IL-10 according to nutritional status of patients with IBD.

	IL-6 (pg/mL) Me [Q1–Q3]	IL-1β (pg/mL) Me [Q1–Q3]	IL-10 (pg/mL) Me [Q1–Q3]
BMI [kg/m^2^]			
<18.5	6.1 [5.9–22.3]	2.9 [2.8–3.0]	6.2 [2.3–20.1]
18.5–24.9	5.2 [4.4–6.3]	2.9 [2.3–10.9]	2.3 [1.9–19.3]
>25	5.3 [3.7–7.7]	2.9 [2.8–13.2]	2.3 [2.1–23.9]
>30	8.7 [5.6–34.3]	6.6 [2.6–25.7]	22.3 [5.1–44.6]
*p*-value	0.1346	0.5881	0.4926
Waist circumference [cm]			
Normal	5.9 [4.4–6.6]	2.9 [2.5–10.9]	2.3 [1.9–19.8]
High	5.5 [3.9–18.8]	3.0 [2.8–14.9]	14.9 [2.1–46.0]
*p*-value	0.4727	0.2627	0.1499
Weight reduction in 6 months [%]			
<10	4.9 [3.8–6.7]	2.9 [2.3–3.1]	2.3 [1.9–20.8]
>10	5.9 [4.9–22.3]	3.0 [2.8–37.4]	3.5 [2.2–23.9]
*p*-value	0.0479 * 0.1445 **	0.0486 * 0.0052 **	0.3108
Adipose tissue [%]			
Low	5.7 [4.4–7.7]	3.0 [2.8–26.2]	8.0 [2.3–20.6]
Normal	4.6 [3.4–5.9]	2.8 [2.3–3.0]	2.4 [1.9–19.8]
High	6.2 [5.9–23.9	2.9 [2.8–13.2]	2.3 [2.0–42.7]
*p*-value	0.0084 * 0.0466 **	0.2662	0.3960
Water content [%]			
Low	5.4 [4.4–7.4]	2.9 [2.5–12.2]	2.3 [1.9–23.6]
Normal	5.9 [3.8–22.3]	2.9 [2.7–26.2]	4.4 [2.2–20.6]
*p*-value	0.7370	0.6796	0.3371

* Univariate analyses. Post-hoc multiple comparisons. Il-6, low vs. normal *p* = 0.0102; low vs. high *p* = 0.0078; normal vs. high *p* = 0.0415. ** Multivariate analyses involved all the above-mentioned factors, stratifying by CD vs. UC and controlling for age and gender.

**Table 7:** Spearman’s correlations coefficients for the investigated cytokines in the study cohort.

	IL-6 (pg/mL)	IL-1β (pg/mL)
All participants		
IL-6 (pg/mL)	-	-
IL-1β (pg/mL)	0.3238 **	-
IL-10 (pg/mL)	0.2616 *	0.3308 **
CD		
IL-6 (pg/mL)	-	-
IL-1β (pg/mL)	0.3155 *	-
IL-10 (pg/mL)	0.4129 **	0.3914 **
UC		
IL-6 (pg/mL)	-	-
IL-1β (pg/mL)	0.3372	-
IL-10 (pg/mL)	0.0323	0.2762

* *p* < 0.001. ** *p* < 0.0001.

## Data Availability

Not applicable.
